# Supplementary material for: Systems Metabolic Engineering of Saccharomyces cerevisiae for the High-Level Production of (2S)-Eriodictyol
Source: J Fungi (Basel). 2024 Jan 31;10(2):119. doi: 10.3390/jof10020119 (PMC10890390; doi:10.3390/jof10020119)
Supplement: Supplementary file 1 [file jof-10-00119-s001.zip › jof-2793836-supplementary.pdf]

Supplemental Materials

Document S1:

Supplementary Figures

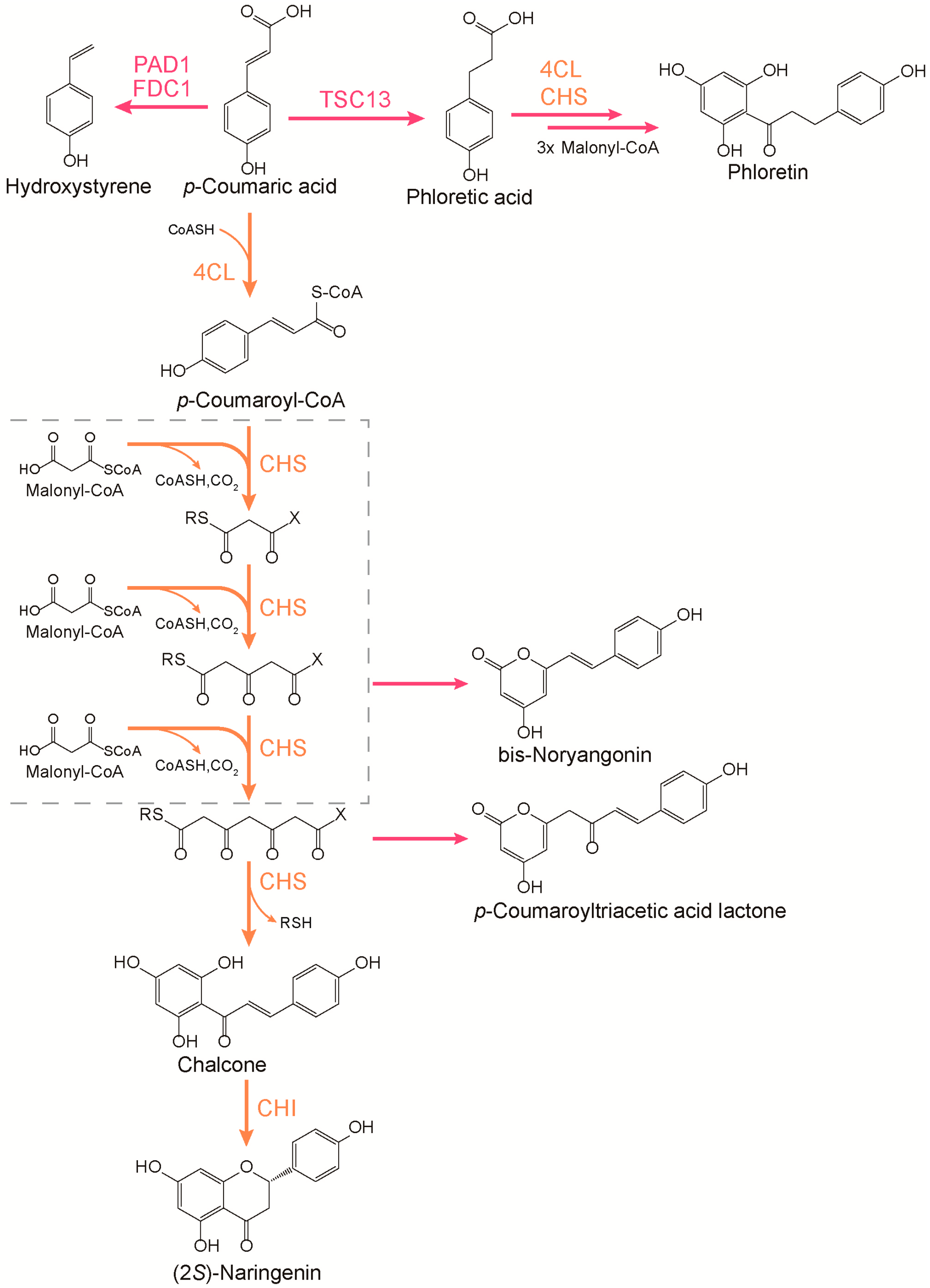

Figure S1. Synthesis of by-products during (2*S*)-naringenin synthesis. Pad1, Phenylacrylic acid decarboxylase; Fdc1, Ferulic acid decarboxylase; Tsc13, Enoyl reductase.

Supplementary Tables

Tables

Table S1. Strains used in this study

Strains	Genotype	Source
S. cerevisiae CEN.PK2-1D	MATα, *ura3-52*, *trp1-289, leu2-3,112*, *his3Δ1*, *MAL2-8C*, *SUC2*	[1]
C800	*CEN.PK2-1D*, *gal80::kanMX*	[1]
SQ01	C800, XI-2::(GAL7p-FjTAL-DIT1t)	This study
SQ02	C800, XI-2::(INO1p-FjTAL-DIT1t)	This study
SQ03	C800, XI-5::(CYC1t-AtC4H-TDH1p)+(PKG1p-AtPAL2-CIT2t)+(SDH1t-AtATR2-TDH3p)+(SED1p-CYB5-PDB1t)	This study
SQ04	C800, XI-5::(CYC1t-AtC4H-GAL10p)+(GAL1p-AtPAL2-CIT2t) +(SDH1t-AtATR2-TDH3p)+(SED1p-CYB5-PDB1t)	This study
SQ05	C800, XI-5::(CYC1t-AtC4H-GAL10p)+(GAL1p-AtPAL2-CIT2t) +(SDH1t-AtATR2-TDH3p)+(SED1p-CYB5-PDB1t), XI-2::(GAL7p-FjTAL-DIT1t)	This study
N01	C800, pY26-ADH1t-PhCHS-GAL10p-CCW12p-MsCHI-ADH2t-SED1p-Pc4CL-CYC1t	This study
N02	C800, pY26-ADH1t-PhCHS-CCW12p-FBA1p-MsCHI-ADH2t-GAL10p-Pc4CL-CYC1t	This study
N03	C800, pY26-ADH1t-PhCHS-GAL10p-FBA1p-MsCHI-ADH2t-SED1p-Pc4CL-CYC1t	This study
N04	C800, pY26-ADH1t-PhCHS-GAL10p-GAL1p-MsCHI-ADH2t-SED1p-Pc4CL-CYC1t	This study
N05	C800, pY26-ADH1t-PhCHS-GAL10p-CCW12p-MsCHI-ADH2t-TDH1p-Pc4CL-CYC1t	This study
N06	C800, pY26-ADH1t-PhCHS-FBA1p-PGK1p-MsCHI-ADH2t-TDH1p-Pc4CL-CYC1t	This study
N07	C800, pY26-ADH1t-PhCHS-GAL10p-GAL1p-MsCHI-ADH2t- GAL7p -Pc4CL-CYC1t	This study
N08	C800,	This study

	pY26-ADH1t-PhCHS-GAL10p-ALD5p-MsCHI-ADH2t-ARO7p-Pc4CL-CYC1t	
N09	C800, pY26-ADH1t-PhCHS-ERG20p-PGK1p-MsCHI-ADH2t-SED1p-Pc4CL-CYC1t	This study
N10	C800, pY26-ADH1t-PhCHS-GAL10p-PGK1p-MsCHI-ADH2t-ARO7p-Pc4CL-CYC1t	This study
N11	C800, pY26-ADH1t-PhCHS-TDH1p-PGK1p-MsCHI-ADH2t-SED1p-Pc4CL-CYC1t	This study
N12	C800, pY26-ADH1t-PhCHS-TDH1p-PGK1p-MsCHI-ADH2t-ARO7p-Pc4CL-CYC1t	This study
N13	C800, pY26-ADH1t-PhCHS-TDH1p-PGK1p-MsCHI-ADH2t-MET6p-Pc4CL-CYC1t	This study
N14	C800, pY26-ADH1t-PhCHS-GAL10p-FBA1p-MsCHI-ADH2t-SED1p-Pc4CL-CYC1t	This study
N15	C800, pY26-ADH1t-PhCHS-ERG20p-FBA1p-MsCHI-ADH2t-SED1p-Pc4CL-CYC1t	This study
N16	C800, pY26-ADH1t-PhCHS-CCW12p-PGK1p-MsCHI-ADH2t-SED1p-Pc4CL-CYC1t	This study
N17	C800, pY26-ADH1t-PhCHS-GAL10p-CCW12p-MsCHI-ADH2t-TDH1p-Pc4CL-CYC1t	This study
N18	C800, pY26-ADH1t-PhCHS-FBA1p-CCW12p-MsCHI-ADH2t-TDH1p-Pc4CL-CYC1t	This study
N19	C800, pY26-ADH1t-PhCHS-GAL10p-PGK1p-MsCHI-ADH2t-SED1p-Pc4CL-CYC1t	This study
N20	C800, pY26-ADH1t-PhCHS-GAL10p-TDH1p-MsCHI-ADH2t-SED1p-Pc4CL-CYC1t	This study
N21	C800, pY26-ADH1t-PhCHS-GAL10p-PGK1p-MsCHI-ADH2t-MET6p-Pc4CL-CYC1t	This study

N22	C800, pY26-ADH1t-PhCHS-GAL10p-TDH1p-MsCHI-ADH2t-MET6p-Pc4CL-CYC1t	This study
N23	SQ05, pY26-ADH1t-PhCHS-GAL10p-ALD5p-MsCHI-ADH2t-ARO7p-Pc4CL-CYC1t	This study
N24	SQ05, pY26-ADH1t-PhCHS-GAL10p-ALD5p-MsCHI-ADH2t-ARO7p-Pc4CL-Tsynth27	This study
N25	SQ05, pY26-ADH1t-PhCHS-GAL10p-ALD5p-MsCHI-ADH2t-ARO7p-Pc4CL-HOG1t	This study
N26	SQ05, pY26-SynTer8-PhCHS-GAL10p-ALD5p-MsCHI-ADH2t-ARO7p-Pc4CL-CYC1t	This study
N27	SQ05, pY26-CPS1t-PhCHS-GAL10p-ALD5p-MsCHI-ADH2t-ARO7p-Pc4CL-CYC1t	This study
N28	SQ05, pY26-ADH1t-PhCHS-GAL10p-ALD5p-MsCHI-T-1300stem6-ARO7p-Pc4CL-CYC1t	This study
N29	SQ05, pY26-ADH1t-PhCHS-GAL10p-ALD5p-MsCHI-DIT1t-ARO7p-Pc4CL-CYC1t	This study
N30	SQ05, pY26-CPS1t-PhCHS-GAL10p-ALD5p-MsCHI-ADH2t-ARO7p-Pc4CL-HOG1t	This study
N31	SQ05, pY26-CPS1t-PhCHS-GAL10p-ALD5p-MsCHI-ADH2t-ARO7p-Pc4CL-CYC1t, PRS424-SED1p-ThF3'H- TPS1t	This study
N32	SQ05, pY26-CPS1t-PhCHS-GAL10p-ALD5p-MsCHI-ADH2t-ARO7p-Pc4CL-CYC1t, PRS424-SED1p-ThF3'H- TPS1t, PRS425-SED1p-ThF3'H- TPS1t	This study

Table S2. Plasmids used in this study

Plasmids	Source
pY26-ADH1t-PhCHS-GAL10p-CCW12p-MsCHI-ADH2t-SED1p-Pc4CL-CYC1t	This study
pY26-ADH1t-PhCHS-CCW12p-FBA1p-MsCHI-ADH2t-GAL10p-Pc4CL-CYC1t	This study
pY26-ADH1t-PhCHS-GAL10p-FBA1p-MsCHI-ADH2t-SED1p-Pc4CL-CYC1t	This study
pY26-ADH1t-PhCHS-GAL10p-GAL1p-MsCHI-ADH2t-SED1p-Pc4CL-CYC1t	This study
pY26-ADH1t-PhCHS-GAL10p-CCW12p-MsCHI-ADH2t-TDH1p-Pc4CL-CYC1t	This study
pY26-ADH1t-PhCHS-FBA1p-PGK1p-MsCHI-ADH2t-TDH1p-Pc4CL-CYC1t	This study
pY26-ADH1t-PhCHS-GAL10p-GAL1p -MsCHI-ADH2t- GAL7p -Pc4CL-CYC1t	This study
pY26-ADH1t-PhCHS-GAL10p-ALD5p-MsCHI-ADH2t-ARO7p-Pc4CL-CYC1t	This study
pY26-ADH1t-PhCHS-ERG20p-PGK1p-MsCHI-ADH2t-SED1p-Pc4CL-CYC1t	This study
pY26-ADH1t-PhCHS-GAL10p-PGK1p-MsCHI-ADH2t-ARO7p-Pc4CL-CYC1t	This study
pY26-ADH1t-PhCHS-TDH1p-PGK1p-MsCHI-ADH2t-SED1p-Pc4CL-CYC1t	This study
pY26-ADH1t-PhCHS-TDH1p-PGK1p-MsCHI-ADH2t-ARO7p-Pc4CL-CYC1t	This study
pY26-ADH1t-PhCHS-TDH1p-PGK1p-MsCHI-ADH2t-MET6p-Pc4CL-CYC1t	This study
pY26-ADH1t-PhCHS-GAL10p-FBA1p-MsCHI-ADH2t-SED1p-Pc4CL-CYC1t	This study
pY26-ADH1t-PhCHS-ERG20p-FBA1p-MsCHI-ADH2t-SED1p-Pc4CL-CYC1t	This study
pY26-ADH1t-PhCHS-CCW12p-PGK1p-MsCHI-ADH2t-SED1p-Pc4CL-CYC1t	This study
pY26-ADH1t-PhCHS-GAL10p-CCW12p-MsCHI-ADH2t-TDH1p-Pc4CL-CYC1t	This study
pY26-ADH1t-PhCHS-FBA1p-CCW12p-MsCHI-ADH2t-TDH1p-Pc4CL-CYC1t	This study
pY26-ADH1t-PhCHS-GAL10p-PGK1p-MsCHI-ADH2t-SED1p-Pc4CL-CYC1t	This study
pY26-ADH1t-PhCHS-GAL10p-TDH1p-MsCHI-ADH2t-SED1p-Pc4CL-CYC1t	This study
pY26-ADH1t-PhCHS-GAL10p-PGK1p-MsCHI-ADH2t-MET6p-Pc4CL-CYC1t	This study
pY26-ADH1t-PhCHS-GAL10p-TDH1p-MsCHI-ADH2t-MET6p-Pc4CL-CYC1t	This study
pY26-ADH1t-PhCHS-GAL10p-ALD5p-MsCHI-ADH2t-ARO7p-Pc4CL-Tsynth27	This study
pY26-ADH1t-PhCHS-GAL10p-ALD5p-MsCHI-ADH2t-ARO7p-Pc4CL-HOG1t	This study
pY26-SynTer8-PhCHS-GAL10p-ALD5p-MsCHI-ADH2t-ARO7p-Pc4CL-CYC1t	This study
pY26-CPS1t-PhCHS-GAL10p-ALD5p-MsCHI-ADH2t-ARO7p-Pc4CL-CYC1t	This study
pY26-ADH1t-PhCHS-GAL10p-ALD5p-MsCHI-T-1300stem6-ARO7p-Pc4CL-CYC1t	This study
pY26-ADH1t-PhCHS-GAL10p-ALD5p-MsCHI-DIT1t-ARO7p-Pc4CL-CYC1t	This study
pY26-CPS1t-PhCHS-GAL10p-ALD5p-MsCHI-ADH2t-ARO7p-Pc4CL-HOG1t	This study
PRS424-SED1p-ThF3'H- TPS1t	This study
PRS425-SED1p-ThF3'H- TPS1t	This study

Table S3. Primers used in this study

Primers	Sequence (5'-3')
X-2 us-F	CGTCTATGAGGAGACTGTTAGTTGG
X-2 us-R	GACCACTTCGAGAGCAAGTTGC
GAL7p-F	CAATGTGCCATAAACTCCGTGCACCATTTGCCAGCTTACTATCCTTCTTG
GAL7p-R	CTCAAGTATTCATTAATGGTGTTCATTTTTGAGGGAATATTCAACTGTTTTTT T
FjTAL-F	ATGAACACCATTAATGAATACTTGAG
FjTAL-R	TTAATTGTTAATCAAATGATCCTTAACC
DIT1t-F	GGTTAAGGATCATTTGATTAACAATTAAATAAAGTAAGAGCGCTACATTGG

DIT1t-R	TATGAAGCAGCCAATACCTGTTTTACAAACTTGTTACTCCGCAACGCTTTTC
INO1-F	CAATGTGCCATAAACTCCGTGCACCAGAAGACGATGAGGCCGGTGC
INO1-R	CTCAAGTATTCATTAATGGTGTTCATTGTTACTTCTTTTTCACTGGAAAAAA AAGG
XI-5 us-F	GCGGAGAAGTCGTTGATAGCATTTC
XI-5 us-R	TGGTGCACGGAGTTTATGGCAC
XI-5 ds-F	TTGTAAAACAGGTATTGGCTGC
XI-5 ds-R	GATCATAGATCCGGCACTTAGAG
CYC1t-R	CAATGTGCCATAAACTCCGTGCACCAGCAAATTAAAGCCTTCGAGCGTC
CYC1t-F	TATTGTTATGAAGCCAAGAAACTGTTAAACAGGCCCCTTTTCCTTTGTCGA
AtC4H-F	TTAACAGTTTCTTGGCTTCATAACAATAATAG
AtC4H-R	ATGGATTTGTTGTTGTTGGAAAAGTC
TDH1p-R	AGACTTTTCCAACAACAACAAATCCATTTGTTTGTGTGTAAATTTAGTGA AGTACTG
TDH1p-F	AGGGAAACACTTCCTTTTTCTGGCCCTGAAACCACACCGTGGGGCCTTG
PGK1p-F	GGGCCAGAAAAAGGAAGTGTTTC
PGK1p-R	CAACATGGCTTCAATTTGATCCATTGTTTTATATTTGTTGTAAAAAGTAGATA ATTAC
AtPAL2-F	ATGGATCAAATTGAAGCCATGTTG
AtPAL2-R	GGGGTGTAAAAGTAGGATGTAATCCAAATTTAACAAATTGGAATTGGAGCA CCG
CIT2t-F	TTGGATTACATCCTACTTTTACACCC
CIT2t-R	GCCGGAATAGTGCAAATTGTATG
SDH1t-R	GATTCATACAATTTGCACTATTCCGGCGACAGCACCCTTGTACAGCAATTC
SDH1t-F	GGTAGATACTTGAGAGATGTTTGGTAATTTGAACCTCATTGTATTTTACGG
AtATR2-F	TTACCAAACATCTCTCAAGTATCTACCG
AtATR2-R	ATGAAGAACATGATGAACTACAAGTTG
TDH3p-R	CAACTTGTAGTTCATCATGTTCTTCATTTTGTTTGTTTATGTGTGTTTATTCGA AAC
TDH3p-F	AGCAATCAGTGGTGTATGGAAGGTAGAATAAAAAACACGCTTTTTCAGTTC GAG
SED1-F	TCTACCTTCCATACACCACTGATTG
SED1-R	CATCTTAATAGAGCGAACGTATTTTATTTTG
CYB5-F	AATAAAATACGTTCGCTCTATTAAGATGATGCCTAAAGTTTACAGTTACCAA G
CYB5-R	CTCTCCTTCCTATTGGATTGAAGTTTTATTCGTTCAACAAATAATAAGCAAC AC
PDB1t-F	AACTTCAATCCAATAGGAAGGAGAG
PDB1t-R	ATGAAGCAGCCAATACCTGTTTTACAACTTTCGCTTAGGTTCCATGCC
GAL1,10p-R	AGACTTTTCCAACAACAACAAATCCATTTATATTGAATTTTCAAAAATTCTT ACTTTTT
GAL1,10p-F	ACAACATGGCTTCAATTTGATCCATTATAGTTTTTTCTCCTTGACGTTAAAGT ATAG
pRS424-F	TATAGCATGAGGTCGCTCAGCTCCAGCTTTTGTTCCCTTTAG
ADH1t-R	AACAAAAGCTGGAGCTGAGCGACCTCATGCTATACC
PhCHS-F	GTTCTGAAACTGTCTCCCCAGCTTTGGATGGTTACGGTGGAAGAATACC
CCW12-F	GCTGGCTATTGGCGTCTGATTTC

PGK1p-F	CGGAAATCAGACGCCAATAGCCAGCGGGCCAGAAAAAGGAAGTGTTTC
Pc4CL-R	AAAAAGTAAGAATTTTTGAAAATTCAATATAAATGGGTGACTGCGTTGCCCCG
FBA1p-F	CGGAAATCAGACGCCAATAGCCAGCATAACAATACTGACAGTACTAAATAATTGC
ADH2t-R	CTATACTTTAACGTCAAGGAGAAAAAACTATAATGAGAAATATCGAGGGAGACGA
FBA1p-F	TACTTTAACGTCAAGGAGAAAAAACTATAATAACAATACTGACAGTACTAAATAATTGC
PhCHS-F	CATATATAACCATAACCAAGTAATACATATTCAAAATGGTTACGGTGGAAGAATACC
FBA1p-F	AACGGAAATCAGACGCCAATAGCCAGCATAACAATACTGACAGTACTAAATAATTGC
FBA1p-F	GAGGGAAACACTTCCTTTTTCTGGCCCATAACAATACTGACAGTACTAAATAATTGC
GAL1,10p-1F	TATAGTTTTTTCTCCTTGACGTTAAAG
GAL1,10p-1R	TTATATTGAATTTTCAAAAATTCTTACTTTTT
CCW12-R	CCAAAGCTGGGGAGACAGTTTC
CCW12-F	GCTGGCTATTGGCGTCTGAT
PGK1p-F	CAGACGCCAATAGCCAGCACGCACAGATATTATAACATCTGC
FBA1p-F	TGTTATAATATCTGTGCGTATAACAATACTGACAGTACTAAATAATTGC
PGK1p-F	ACGCACAGATATTATAACATCTGC
ADH2t-R	GGATAGTAAGCTGGCAAAATGAGAAATATCGAGGGAGACGA
GAL7p-F	TTTGCCAGCTTACTATCCTTCTTG
GAL7p-R	TTTTGAGGGAATATTCAACTGTTTTTTT
Pc4CL-R	GTTGAATATTCCCTCAAAAATGGGTGACTGCGTTGCC
PhCHS-F	ATGGTTACGGTGGAAGAATACCG
Pc4CL-F	ATGGGTGACTGCGTTGCC
MsCHI-F	ATGGCAGCAAGCATTACGGC
JID1t-R	ATCTTATACCAATTTTATGCAGGATGCTG
ERG20p-F	GTTATAATATCTGTGCGTCTCACACTTTGGTTCTTCGAC
PGK1p-R	CGTAATGCTTGCTGCCATTTATATTTGTTGTAAAAAGTAGATAATTAC
SED1-F	GCATAAAATTGGTATAAGATTCTACCTTCCATACACCACTGATTG
SED1-R	GCAACGCAGTCACCCATCTTAATAGAGCGAACGTATTTTATTTTGC
PGK1p-F	GTCAAGGAGAAAAAACTATAACGCACAGATATTATAACATCTGC
TDH1p-R	TTCTTCCACCGTAACCATTTTGTTTTGTGTGTAAATTTAGTGAAG
TDH1p-F	GTTATAATATCTGTGCGTTGAAACCACACCGTGGGG
MET6p-F	GCATAAAATTGGTATAAGATCATGAACCAGGGTCCCGCAC
MET6p-R	GCAACGCAGTCACCCATTTTTGATATGTACTTTGAAATTATATTGG
FBA1p-R	GTAATGCTTGCTGCCATTTTGAATATGTATTACTTGGTTATGG
ERG20p-F	GTACTGTCAGTATTGTTATCTCACACTTTGGTTCTTCGAC
FBA1p-F	ATAACAATACTGACAGTACTAAATAATTGC
CCW12p-R	CTTCCACCGTAACCATCCAAAGCTGGGGAGACAGTTTC
CCW12p-R	GTAATGCTTGCTGCCATCCAAAGCTGGGGAGACAGTTTC

TDH1p-F	GCATAAAATTGGTATAAGATTGAAACCACACCGTGGGGC
TDH1p-R	GCAACGCAGTCACCCATTTTGTTTTGTGTGTAAATTTAGTGAAG
FBA1p-R	TTCTTCCACCGTAACCATTTTGAATATGTATTACTTGGTTATGG
FBA1p-F	GACGCCAATAGCCAGCATAACAATACTGACAGTACTAAATAATTGC
TDH1p-F	GTCAAGGAGAAAAAACTATATGAAACCACACCGTGGGG
TDH1p-R	GTAATGCTTGCTGCCATTTTGTTTTGTGTGTAAATTTAGTGAAGTAC
pRS424C-F	GTCTCCCTCGATATTTCTCATAGCTCCAGCTTTTGTTCCCTTTAG
pRS424C-R	CCTGCCGAAGTAATCATGTAATTAGTTATGTCACGCTTACATTC
Pc4CL-F	GTGACATAACTAATTACATGATTACTTCGGCAGGTCGCCGCTCG
Pc4CL-R	AATACGTTCGCTCTATTAAGATGGGTGACTGCGTTGCCCCG
SED1-R	GTCACCCATCTTAATAGAGCGAACGTATTTTATTTTGC
SED1-F	CAAAGTGTGAGTCTACCTTCCATACACCACTGATTG
ERG20-F	GTGGTGTATGGAAGGTAGACTCACACTTTGGTTCTTCGAC
ERG20-R	ATTCTTCCACCGTAACCATTTCTACGTAATATTTTTGAGTTTATTGTGC
PhCHS-F	ATTACGTAGAAATGGTTACGGTGGAAGAATACC
PhCHS-R	AAATCATAAGAAATTCGCTTAGGTAGCCACACTATGCAG
ADH1t-F	GTGGCTACCTAAGCGAATTTCTTATGATTTATGATTTTTATT
ADH1T-R	TTCCTTTTTCTGGCCCGAGCGACCTCATGCTATACC
PGK1p-F	ATGAGGTCGCTCGGGCCAGAAAAAGGAAGTGTTTC
PGK1p-R	AATGCTTGCTGCCATTGTTTTATATTTGTTGTAAAAAGTAGATAATTAC
MsCHI-F	CAACAAATATAAAACAATGGCAGCAAGCATTACGGC
MsCHI-R	GACATAAGAGATCCGCTCAGTTACCGATTTTAAAGGCACCTTC
ADH2t-F	AAATCGGTAACTGAGCGGATCTCTTATGTCTTTACG
ADH2t-R	GCTGGAGCTATGAGAAATATCGAGGGAGACGA
DIT1t-F	CGGTAACTGATAAAGTAAGAGCGCTACATTGGTC
DIT1-R	CTGGAGCTTTACTCCGCAACGCTTTTCTG
SDIT1t-F	TTGCGGAGTAAAGCTCCAGCTTTTGTTCCCTTTAG
SDIT1-R	TCTTACTTTATCAGTTACCGATTTTAAAGGCACCTTC
T-1300 stem 6-F	CTTGTGGAAAAAAGGGGGAAAGGGCCCCCTTTCAAAAGCTCCAGCTTTTG TTCCCTTTAG
T-1300 stem 6-R	TTTCCCCCTTTTTTCCACAAGGGATATATATATATATATATATATATATCAGTTA CCGATTTTAAAGGCACCTTC
CPS1t-F	CTACCTAAGCGCAATGATTGAATAGTCAAAG
CPS1t-R	TCTGGCCCATTTGACACTTGATTTGACACTTC
SCPS1t-F	CAAGTGTCAAATGGGCCAGAAAAAGGAAGTGTTTC
SCPS1t-R	AATCATTGCGCTTAGGTAGCCACACTATGCAG
SynTer8-F	TAAAGACGTTGAGTCCGTTGAGAGTATCATCGAGTATCATCTTTCAAAGGG CCAGAAAAAGGAAGTGTTTC
SynTer8-R	CAACGGACTCAACGTCTTTATTTCTAGACAGTTATATATTAGGTAGCCACAC TATGCAG
HOG1t-F	AGTAATTTAATGTCCCTAACCACTCATTC
HOG1t-R	GGTACCGGCCTACGCTTGTCTGGGTGAGAC
SHOG1t-F	ACAAGCGTAGGCCGGTACCCAATTCGCCC
SHOG1t-R	GTTAGGGACATTAAATTACTTCGGCAGGTCGCCGCTCG
Tsynth27-F	AACTGTCTAGAAATAAAGAGTATCATCTTTCAAAGGCCGGTACCCAATTCG CC

Tsynth27-R	ACTCTTTATTTCTAGACAGTTATATATATATATATATATATATATACCACCCATTA CTTCGGCAGGTCGCCGC
SED1p-R	CTTAATAGAGCGAACGTATTTTATTTTGCTTGTCT
ThF3'H-F	AATAAAATACGTTCGCTCTATTAAGATGAGCATCCTGATCATCATCATCCT
ThF3'H-R	GATCGTCTCATTTGCATCGGGTTCATTAGCATTCAACAACTTCATACGCACC
TPS1t-F	TGAACCCGATGCAAATGAGACG
URA3-F	GCAGATTGTACTGAGAGTGCACC
URA3-R	ACGCATCTGTGCGGTATTTCAC
Prs425-F	GAAATACCGCACAGATGCGTAAG
Prs425-R	GCACTCTCAGTACAATCTGCTC

Table S4. Exogenous genes used in this study

Exogenous gene	NCBI Reference Sequence	Enzyme	Organism	Reference
FjTAL	WP_012023194.1	tyrosine ammonia-lyase	*Flavobacterium johnsoniae*	[1]
AtPAL2	NP_190894.1	phenylalanine ammonia-lyase 2	*Arabidopsis thaliana*	[1]
AtC4H	NP_180607.1	cinnamate-4-hydroxylase	*Arabidopsis thaliana*	[1]
AtATR2	NP_001328167.1	P450 reductase 2	*Arabidopsis thaliana*	[1]
Pc4CL	P14912.1	4-coumaroyl-CoA ligase	*Petroselinum crispum*	[1]
PhCHS	AAF60297.1	chalcone synthase	*Petunia x hybrida*	[1]
MsCHI	P28012.1	Chalcone isomerase 1	*Medicago sativa*	[1]
ThF3'H	BAH22519.1	flavonoid 3' hydroxylase	*Tricyrtis hirta*	[2]

Table S5. Natural strong terminator and artificial strong terminator sequence

Terminator	Sequence (5'-3')	Source
Tsynth27	TGGGTGGTATATATATATATATATATATATATATAACTGTCTAGAAATAAAG AGTATCATCTTTCAAA	[3]
HOG1t	TTTAATGTCCCTAACCACTCATTCTTACTTCTTTTTGATGTTTCTTTTT TTTATGGTACTCATAAAAGTATTTACGTATATAGTTGTATAGAGGAAA CAAAAAAAAAAAGATAAAACTCAATTACAAAGTAAAGTGGACGTA TTTCGATCATGATTTTTTTCTGTTTTAACCGCATTGGATTTTCTTGTA AAACTGGAAGAAAAAGGAAACTAAAAAGTCAAGAAAGACCTTTTT AAGACTCCAAGAACCGTCACTTATGGCGTATTGTTTGTTTATCAGCA CTTCTATCTTCGATAAAGGTTTGTCTGTCTTATATTGTTTACATTTCAA GTCTAATTCTGTGCTTTTACCGAAGAGGAATTTTCATAAATACGGAG AAAATATAAAAAAAAAGTAAATACAGAAAATAGAACAGTTGAAGC AGAAAAAGAGAACTTGCTAAATAGCTGTCTCACCCAGACAAGCGT	[4]

	A	
SynTer8	TATATATATATAACTGTCTAGAAATAAAAATAAAGATGATGAGAGTAT<br>CATCTTTCAAATTTCAAA	[5]
CPS1t	GCGCAATGATTGAATAGTCAAAGATTTTTTTTTTTAATTTTTTTTTT<br>TTAATTTTTTTTTTTTCATAGAACTTTTTATTTAAATAAATCACGTC<br>TATATATGTATCAGTATAACGTAAAAAAAAAAACACCGTCAGTTAAA<br>CAAAACATAAATAAAAAAAAAAAGAAGTGTCAAATCAAGTGTCAA<br>AT	[6]
T-1300 stem 6	TATATATATATATATATATATATCCCTTGTGGAAAAAAGGGGGAAAG<br>GGCCCCCTTTCAAA	[7]
DIT1t	TAAAGTAAGAGCGCTACATTGGTCTACCTTTTTGTTCTTTTACTTAA<br>ACATTAGTTAGTTCGTTTTCTTTTTCTCATTTTTTTATGTTTCCCCCCC<br>AAAGTTCTGATTTTATAATATTTTATTTCACACAATTCCATTTAACAG<br>AGGGGGAATAGATTCTTTAGCTTAGAAAATTAGTGATCAATATATATT<br>TGCCTTTCTTTTCATCTTTTCAGTGATATTAATGGTTTCGAGACACTG<br>CAATGGCCCTAGTTGTCTAAGAGGATAGATGTTACTGTCAAAGATGA<br>TATTTTGAATTTCAATTGACGTAATTAATGATACTATTAATAATACAGA<br>GCGTATATGAAGTATTGCAAATAACATGCACAGTTCTTTTGGGATGA<br>GAATGATAATGAAAGGCGAAGGCGGGCGTTCAGAAAAGCGTTGCG<br>GAGTAA	[8]

References

1. Gao, S.; Zhou, H.; Zhou, J.; Chen, J. Promoter-Library-Based Pathway Optimization for Efficient (2*S*)-Naringenin Production from *p*-Coumaric Acid in *Saccharomyces Cerevisiae*. J Agric Food Chem. **2020**, 68, 6884-6891.
2. Liu, J.; Xiao, Z.; Zhang, S.; Wang, Z.; Chen, Y.; Shan, Y. Restricting Promiscuity of Plant Flavonoid 3'-Hydroxylase and 4'-*O*-Methyltransferase Improves the Biosynthesis of (2*S*)-Hesperetin in *E. Coli*. J Agric Food Chem. **2023**, 71, 9826-9835.
3. Curran, K.A.; Morse, N.J.; Markham, K.A.; Wagman, A.M.; Gupta, A.; Alper, H.S. Short Synthetic Terminators for Improved Heterologous Gene Expression in Yeast. ACS Synth Biol. **2015**, 4, 824-832.
4. Wei, L.; Wang, Z.; Zhang, G.; Ye, B. Characterization of Terminators in *Saccharomyces Cerevisiae* and an Exploration of Factors Affecting Their Strength. Chembiochem. **2017**, 18, 2422-2427.
5. Ahmed, M.S.; Ikram, S.; Rasool, A.; Li, C. Design and Construction of Short Synthetic Terminators for Β-Amyrin Production in *Saccharomyces Cerevisiae*. Biochem Eng J. **2019**, 146, 105-116.
6. Curran, K.A.; Karim, A.S.; Gupta, A.; Alper, H.S. Use of Expression-Enhancing Terminators in *Saccharomyces Cerevisiae* to Increase Mrna Half-Life and Improve Gene Expression Control for Metabolic Engineering Applications. Metab Eng. **2013**, 19, 88-97.
7. Wang, Z.; Wei, L.; Sheng, Y.; Zhang, G. Yeast Synthetic Terminators: Fine Regulation of Strength Through Linker Sequences. Chembiochem. **2019**, 20, 2383-2389.
8. Ito, Y.; Yamanishi, M.; Ikeuchi, A.; Matsuyama, T. A Highly Tunable System for the Simultaneous Expression of Multiple Enzymes in *Saccharomyces Cerevisiae*. ACS Synth Biol. **2015**, 4, 12-16.
